# Seasonal Variation in Biting Rates of *Simulium damnosum sensu lato*, Vector of *Onchocerca volvulus*, in Two Sudanese Foci

**DOI:** 10.1371/journal.pone.0150309

**Published:** 2016-03-04

**Authors:** Isam M. A. Zarroug, Kamal Hashim, Arwa H. Elaagip, Abdallah M. Samy, Ehab A. Frah, Wigdan A. ElMubarak, Hanan A. Mohamed, Tong Chor M. Deran, Nabil Aziz, Tarig B. Higazi

**Affiliations:** 1 Onchocerciasis Control/Elimination Programme, National Programme for Prevention of Blindness (NPPB), Federal Ministry of Health, Khartoum, Sudan; 2 National Programme for Prevention of Blindness (NPPB), Federal Ministry of Health, Khartoum, Sudan; 3 Department of Parasitology and Medical Entomology, Faculty of Medical Laboratory Sciences, University of Khartoum, Khartoum, Sudan; 4 Entomology Department, Faculty of Science, Ain Shams University, Abbassia, Cairo, Egypt; 5 Biostatistics and Data Analysis Unit, Tropical Medicine Research Institute (TMRI), National Center for Research, Khartoum, Sudan; 6 The Carter Center, Khartoum, Sudan; 7 Department of Biological Sciences, Ohio University, Zanesville, Ohio, United States of America; Fundação Oswaldo Cruz, BRAZIL

## Abstract

**Background:**

The abundance of onchocerciasis vectors affects the epidemiology of disease in Sudan, therefore, studies of vector dynamics are crucial for onchocerciasis control/elimination programs. This study aims to compare the relative abundance, monthly biting-rates (MBR) and hourly-based distribution of onchocerciasis vectors in Abu-Hamed and Galabat foci. These seasonally-based factors can be used to structure vector control efforts to reduce fly-biting rates as a component of onchocerciasis elimination programs.

**Methods:**

A cross-sectional study was conducted in four endemic villages in Abu-Hamed and Galabat foci during two non-consecutive years (2007–2008 and 2009–2010). Both adults and aquatic stages of the potential onchocerciasis vector *Simulium damnosum sensu lato* were collected following standard procedures during wet and dry seasons. Adult flies were collected using human landing capture for 5 days/month. The data was recorded on handheld data collection sheets to calculate the relative abundance, MBR, and hourly-based distribution associated with climatic factors. The data analysis was carried out using ANOVA and Spearman rank correlation tests.

**Results:**

Data on vector surveillance revealed higher relative abundance of *S*. *damnosum s*.*l*. in Abu- Hamed (39,934 flies) than Galabat (8,202 flies). In Abu-Hamed, vector populations increased in January-April then declined in June-July until they disappeared in August-October. Highest black fly density and MBR were found in March 2007 (*N* = 9,444, MBR = 58,552.8 bites/person/month), and March 2010 (*N* = 2,603, MBR = 16,138.6 bites/person/month) while none of flies were collected in August-October (MBR = 0 bites/person/month). In Galabat, vectors increased in September-December, then decreased in February-June. The highest vector density and MBR were recorded in September 2007 (*N* = 1,138, MBR = 6,828 bites/person/month) and September 2010 (*N* = 1,163, MBR = 6,978 bites/person/month), whereas, none appeared in collection from April to June. There was a significant difference in mean monthly density of *S*. *damnosum s*.*l*. across the two foci in 2007–2008 (df = 3, F = 3.91, *P* = 0.011). Minimum temperature showed significant correlation with adult flies counts in four areas sampled; the adult counts were increased in Nady village (*r*_*s*_ = 0.799) and were decreased in Kalasecal (*r*_*s*_ = - 0.676), Gumaiza (*r*_*s*_ = - 0.585), and Hilat Khateir (*r*_*s*_ = - 0.496). Maximum temperature showed positive correlation with black fly counts only in Galabat focus. Precipitation was significantly correlated with adult flies counts in Nady village, Abu-Hamed, but no significance was found in the rest of the sampled villages in both foci. Hourly-based distribution of black flies showed a unimodal pattern in Abu-Hamed with one peak (10:00–18:00), while a bimodal pattern with two peaks (07:00–10:00) and (14:00–18:00) was exhibited in Galabat.

**Conclusion:**

Transmission of onchocerciasis in both foci showed marked differences in seasonality, which may be attributed to ecology, microclimate and proximity of breeding sites to collection sites. The seasonal shifts between the two foci might be related to variations in climate zones. This information on black fly vector seasonality, ecology, distribution and biting activity has obvious implications in monitoring transmission levels to guide the national and regional onchocerciasis elimination programs in Sudan.

## Introduction

Onchocerciasis (river blindness), a parasitic disease caused by a filarial worm, is geographically widespread but is most prevalent in sub-Saharan Africa. The black fly *Simulium* (*Edwardsellum*) *damnosum sensu lato* (Diptera: Simuliidae) transmits the causative agent *Onchocerca volvulus* (Leuckart) (Spirudida: Onchocercidae), which causes blindness and skin pathologies in humans [1]. A global total of 37 million people in 34 endemic countries are currently at risk of infection and about 99% of the global burden is currently occurs in Africa [2, 3]. In eastern Africa, the flies have a wide geographical distribution; however, the disease itself is focal in nature [4]. This observation led to the discovery of many chromosomally distinct entities (cytospecies and cytotypes) within the *S*. *damnosum* complex, which can differ in their host preferences and vectorial capacity to transmit *Onchocerca volvulus* [4, 5].

The disease tends to vary in severity in different ecological zones with cytogenetically different vectors; hence, the division of *S*. *damnosum s*.*l*. into savanna flies, which transmit savanna strain of *O*. *volvulus* that most often causes blindness, and forest flies, which transmit the forest strain of the parasite that causes skin disease as its main pathogenicity rather than an eye disease [6, 7]. The prevalence of human onchocerciasis has been observed to be directly related to the presence and abundance of its vector; therefore, a detailed understanding of parasite and vector population dynamics is crucial for vector control [8] and is considered as a key component in monitoring the transmission level that measures the success of any of the national and regional control programs [9]. Although control of onchocerciasis formerly depended on mass drug administration (Mectizan/DEC/Albendizole) in most African countries, interrupting disease transmission could also be facilitated by reducing human-fly contact (i.e. biting rate parameter in the transmission cycle) [2, 9].

Onchocerciasis transmission varies in Africa according to variety of factors, such as vector species, vector abundances, seasons and habitats [10]. The *S*. *damnosum* complex has been incriminated in the transmission of the onchocerciasis with different capacities in different bioclimatic zones [11]. Many studies revealed the effect of meteorological conditions, such as relative humidity, temperature, wind velocity, light intensity and rainfall on the black fly biting activity [12]. Most of these meteorological factors are inter-dependent, and different conclusions come from these studies [13]. Changes in the distribution patterns of *S*. *damnosum* occur annually in association with dry and wet seasonal climatic changes. Other factors influencing species distribution include the physical and chemical properties of rivers and human activities that change the habitats of black flies, e.g., deforestation [14] and hydropower dams [15].

The current study was designed to obtain data on seasonal distribution and population changes of adult and aquatic stages of the potential black fly vector *S*. *damnosum s*.*l*. in Abu-Hamed and Galabat foci, by comparing the relative abundance, monthly biting-rate [16] and hourly-based distribution of biting-rates during dry and wet seasons in these two climate distinct zones. These zones were selected to represent two natural bioclimatic zones (i.e., desert and savanna types) with distinguishable meteorological variables to help in planning and assessing the progress of the onchocerciasis control/elimination program in both foci.

## Materials and Methods

### Study areas

**Fig 1:**
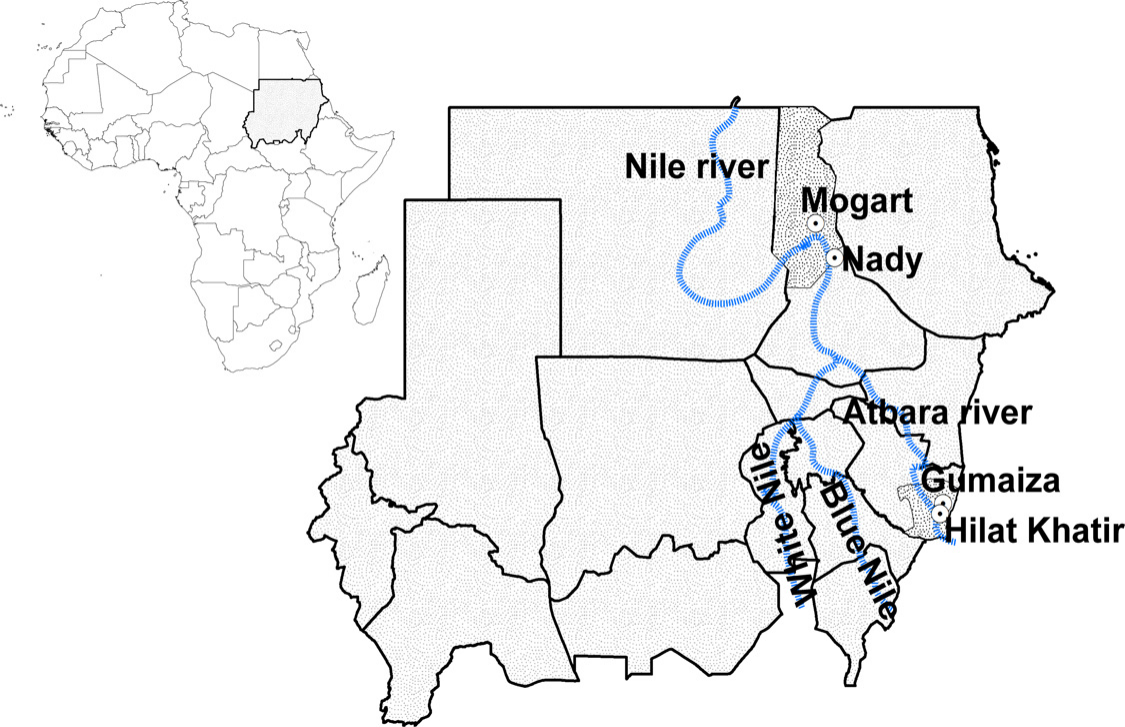
Regional and local map of the study sites in Sudan. The areas highlighted with grey represent the two endemic foci in Abu-Hamed and Galabat. The dotted circles on the right represent the villages selected in each focus.

The Abu-Hamed focus is centered around the River Nile in the middle of the Nubian Desert (N 19° 32.49`–18° 17.29`, E 32° 13.86`–33° 55.00`) at an altitude range of 260–920 km. The breeding of black fly’s aquatic stages lasts from October to May when the level of the Nile is relatively stable [17]. In Abu-Hamed focus, two endemic and sentinel villages of the onchocerciasis control/elimination program near vector breeding sites were selected: Nady village (N 18° 44.46`, E 33° 39.59`, population = 1,168 inhabitants) and Mograt village (N 91°01.99`, E 33° 14.17`, population = 932 inhabitants) [18,19] (Fig 1). Onchocerciasis prevalence in the focus was about 37% in 1985 [20] but transmission of the disease is currently interrupted [19].

The Galabat focus is found in eastern Sudan around the Atbara River (N 14° 06`–12° 57`, E 35° 56`–36° 09`) at an altitude range of 540–1040 km. The water level at Atbara River increases gradually from July to reach its peak from August to October in the rainy season, then decreases rapidly and forms isolated pools from March to May of each year [17]. In Galabat focus, two endemic and sentinel villages of the onchocerciasis control/elimination program near vector breeding sites were selected: Gumaiza (N 13° 25.94`, E 36° 05.70`, population = 3,799 inhabitants) and Hilat Khatir (N 13° 13.10`, E 36° 01.77`, population = 1,914 inhabitants) [18] (Fig 1). The prevalence of onchocerciasis in Galabat focus was reported as 63% in 1987 [21].

### Black fly collection and processing

The aquatic stages of *Simulium* sp. were collected following standard procedures [22] from March 2007 to February 2008 and from November 2009 to October 2010 in Abu-Hamed focus and from June 2007 to May 2008 and June 2009 to May 2010 in Galabat focus. Aquatic black fly stages were collected from submerged plants like *Digitaria ciliaris*, known locally as “Dees” in the fast flowing water of River Nile and the seasonal Atbara River (S1 Fig). During the dry season, aquatic stages were collected only at Galabat focus from *Cynodon dactylon* known locally as “Nageela” in shallow water, and from *Kanahia laniflora* known locally as “Guweer” and *Digitaria ciliaris* in deep water [23]. The collected samples were preserved in 80% alcohol, transferred to the Onchocerciaisis Research Unit, National Public Health Laboratory, Federal Ministry of Health, Sudan for examination and identification using the available morphological keys [24–26]. Pupal exuviae were recorded as an indicator for adult emergence in collection sites.

In each site, adult black flies were collected using the standard human landing capture method [15, 19, 22, 27] for five days a month by four trained volunteers from the local communities who received regular semiannual Ivermectin treatments from 7:00 to 18:00; the local collectors were rotating every three hours during the collection time. The collections were carried out in both foci during the same periods of aquatic stages collections. The collected adult black flies were classified to the species level using the available morphological keys [4, 5, 24, 25, 28].

**Fig 2:**
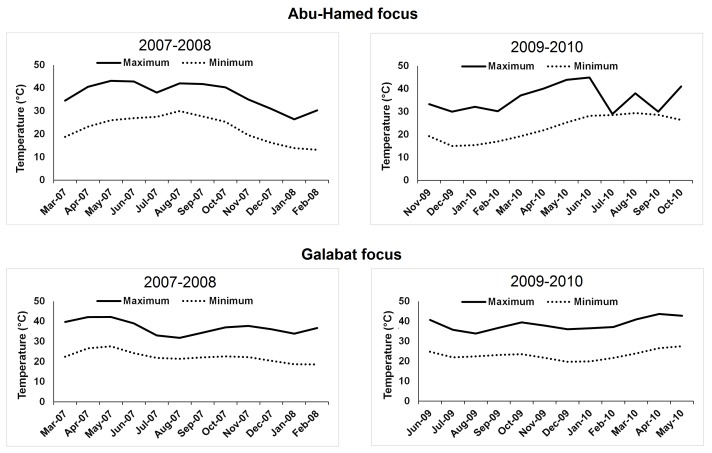
Temperature profile during 2007–2008 and 2009–2010 according to black fly collection activities in Abu-Hamed and Galabat, Sudan.

**Fig 3:**
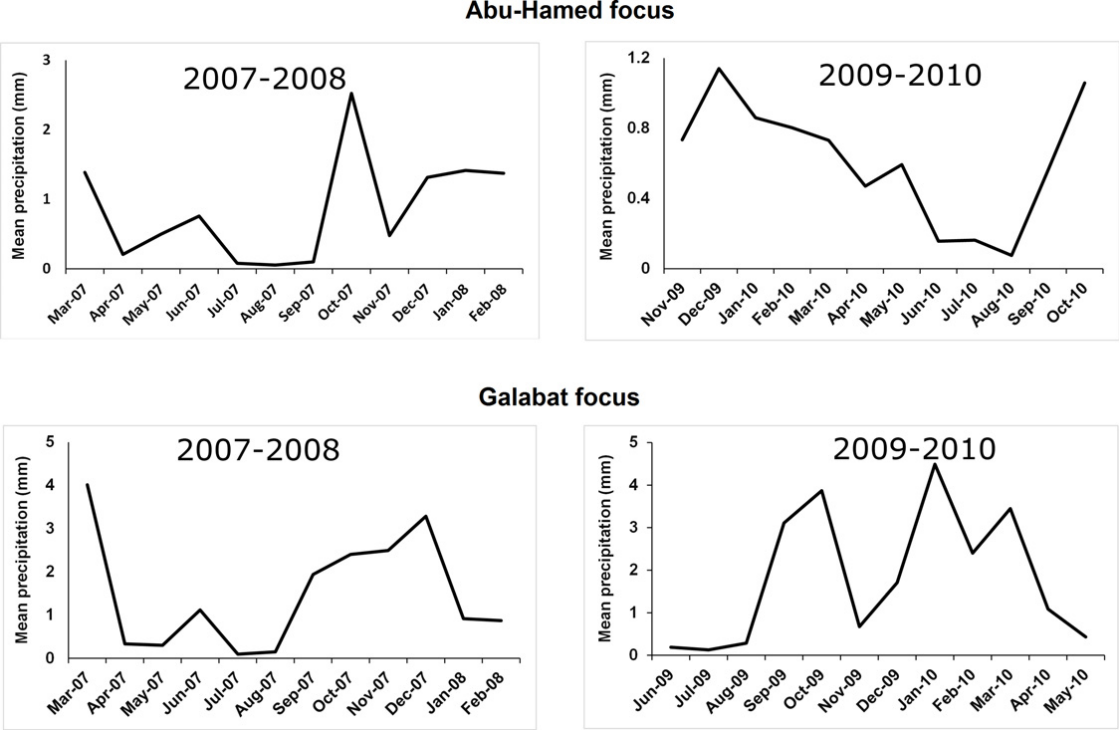
Mean monthly precipitation rates during 2007–2008 and 2009–2010 according to black fly collection activity in Abu-Hamed and Galabat, Sudan.

Maximum and minimum temperatures were obtained from the meteorological data collected by local climatological stations in the study area and deposited in the Weather Online platform (www.weatheronline.co.uk). The rainfall data was collected from 2007 to 2010 from the Global Precipitation Climatology Project’s (GPCP’s) version 2.2 data set. Rainfall values (mm/day) data was sampled from raster data for the sites in the study area using ArcGIS 10.2. All meteorological data were collected to coincide with the same time periods of the study (Fig 2, Fig 3).

Hourly-based black fly distribution was recorded and added to calculate flies biting-rate per man-day [29]. Monthly biting-rate (MBR) was calculated as follows [28]:

$$MBR=\frac{Number\;of\;flies\;caught\times Number\;of\;days\;in\;month}{Number\;of\;catching\;days}$$

### Ethical approval

All study participants received full and detailed information regarding the study procedures and objectives based on their local language. Each participant agreed verbally and signed an informed consent to participate as a volunteer in the study. All volunteers accepted to participate in the study, including black fly collectors, received a quarantine Ivermectin treatment semi-annually as part of the local population. The study was approved by the community leaders, state MOH (River Nile and Gedarif), and the Federal Ministry of Health, Sudan. Ethical approval for the study was provided by the Federal Ministry of Health, Sudan as a part of the national elimination/control programs.

### Data analysis

The data was recorded on handheld data collection sheets before statistical analysis. The ANOVA and Spearman rank correlation tests were used in Minitab software version 16 [30]. ANOVA was used to compare the mean monthly numbers of *S*. *damnosum s*.*l*. across all sampled sites during study periods. The Spearman rank correlation (*Rho*) test was used to assess the correlation between the collected number of flies, maximum temperature, minimum temperature, and precipitation.

## Results

A total of 48,136 adult flies were collected from the two endemic foci in Abu-Hamed and Galabat during two non-consecutive years of 2007–2008 and 2009–2010 (Table 1). A total of 39,934 flies were collected in Abu-Hamed, whereas only 8,202 flies were collected from Galabat focus. The collected adult flies were identified as *S*. *damnosum s*.*l*. and the number of collected flies differed in 2007–2008 and 2009–2010 in both foci (Table 1).

**Table 1:** The number of adult *Simulium damnosum s*. *l*. black flies collected from Abu-Hamed and Galabat foci during 2007–2008 and 2009–2010.

Focus	Total Number of flies collected					
	Abu-Hamed			Galabat		
	Nady	Kalasecal	Total	Gumaiza	Hilat Khateir	Total
**2007–2008**	9521	21510	31031	1561	3043	4604
**2009–2010**	4583	4320	8903	784	2814	3598
**Total**	14104	25830	39934	2345	5857	8202

Collected immature stages revealed seasonal patterns in the two foci sampled. The identification of immature stages in both foci showed three species; *S*. *damnosum s*.*l*. and non-human biting flies (*S*. *griseicollis* and *S*. *adersi*) (Table 2). Immature stages of non-human biting flies were occasionally encountered.

**Table 2:** Seasonal distribution and relative abundance of immature stages of black flies sampled from breeding sites in study areas of Abu-Hamed and Galabat foci during the study period 2007–2008 and 2009–2010.

Black fly species	Abu-Hamed		Galabat	
	Dry Season	Wet Season	Dry Season	Wet Season
***S*. *damnosum s*.*l*.**	**+++**	**+**	+	**+++**
***S*. *griseicollis***	**+**	+	+	**+**
***S*. *adersi***	+	+	+	**+**

Number of (+) represents relative abundance

**Fig 4:**
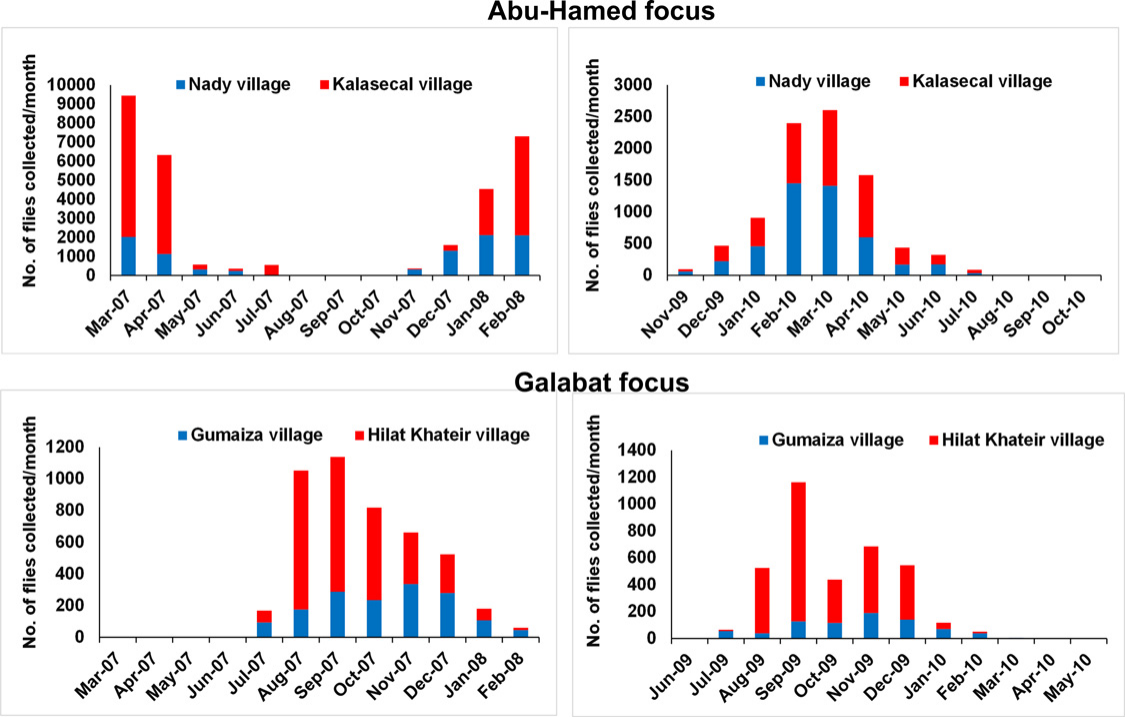
Monthly density of black flies collected in Abu-Hamed and Galabat in 2007–2008 and 2009–2010.

The activity of adult black flies in both foci varied each month. In Abu-Hamed, the host-seeking females started to appear during November, subsequent increased activity during the dry months of January-April, then declined in the beginning of the slightly wet and flooding months (June-July) and disappeared from August to October in 2007–2008 and 2009–2010 (Fig 4).

**Fig 5:**
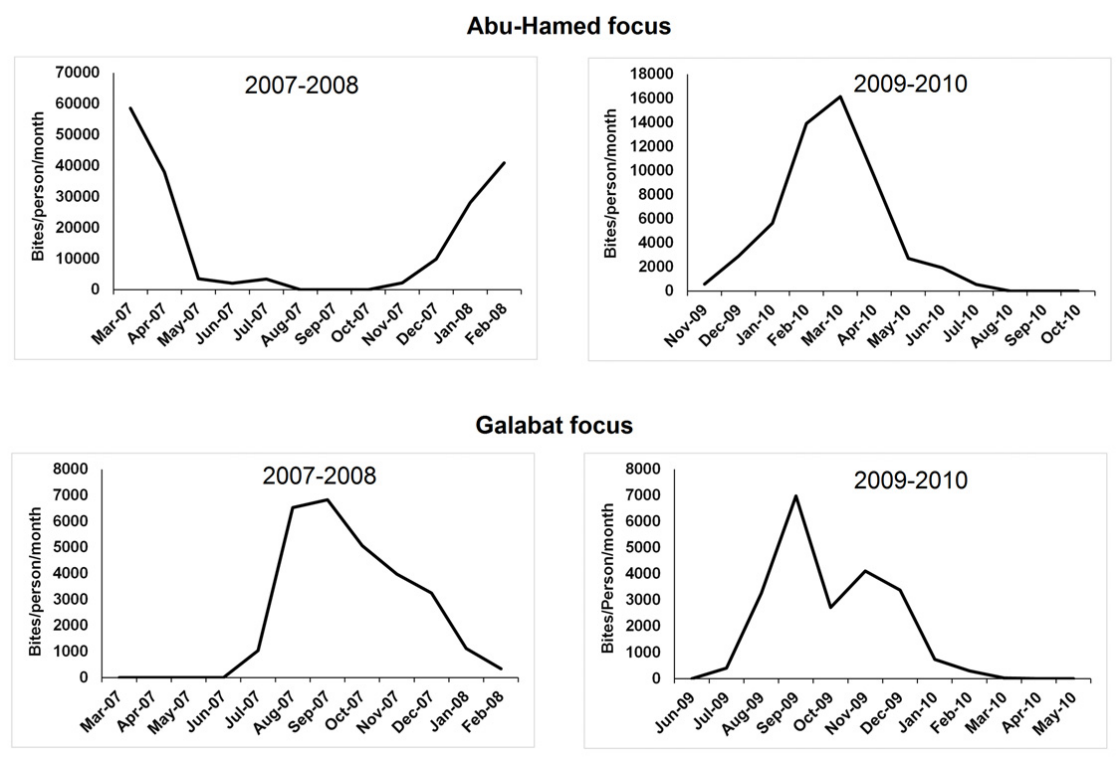
Monthly biting-rate of the host-seeking females collected in Abu-Hamed and Galabat in 2007–2008 and 2009–2010.

In 2007–2008, the highest black fly monthly density (*N* = 9,444) (Fig 4) and peak monthly biting-rate (MBR = 58,552.8 bites/person/month) were found in Abu-Hamed during March 2007 (Fig 5) when the maximum and minimum temperatures were 34.5°C, and 18.8°C, respectively (Fig 2). The lowest monthly density (*N* = 0) (Fig 4) and monthly biting-rate (MBR = 0 bites/person/month) in the same year were found in August through October (Fig 5). In comparison, the highest monthly density (*N* = 2,603) (Fig 4) and peak monthly biting-rate (MBR = 16,138.6 bites/person/month) in 2009–2010 were seen in March 2010 (Fig 5) when maximum, and minimum temperatures were 37.1°C, and 19.3°C, respectively (Fig 2) while the lowest monthly density (*N* = 0) (Fig 4) and monthly biting-rate (MBR = 0 bites/person/month) were found in August through October (Fig 5).

Black flies activity in the Galabat focus appeared in July and increased in the wet months (September through December), then decreased in the dry months (February-June) in both years (Fig 4).

The highest black fly monthly density (*N* = 1,138) (Fig 4) and peak monthly biting-rate (MBR = 6,828 bites/person/month) were found in Galabat during September 2007 (Fig 5) with maximum and minimum temperatures of 34.4°C and 22.1°C respectively (Fig 2). The lowest monthly density (*N* = 0) (Fig 4) and monthly biting-rate (MBR = 0 bites/person/month) were observed during March through June (Fig 5). In 2009–2010, the Galabat focus showed the highest black fly monthly density (*N* = 1,163) (Fig 4) and peak monthly biting-rate (MBR = 6,978 bites/person/month) in September 2009 (Fig 5) with maximum temperature and minimum temperature of 36.7°C and 23.1°C respectively (Fig 2) while the lowest monthly density (*N* = 0) (Fig 4) and monthly biting-rate (MBR = 0 bites/person/month) were found in June 2009, April and May 2010 (Fig 5).

ANOVA analysis showed significant difference in the mean monthly density of *S*. *damnosum s*.*l*. across all sites sampled (df = 3, F = 3.91, *P* = 0.011) in 2007–2008 only. The Spearman rank correlation coefficient revealed significant correlation between minimum temperature and adult fly counts in four areas sampled; the adult counts were increased in Nady village (*r*_*s*_ = 0.799) and were decreased in Kalasecal (*r*_*s*_ = - 0.676), Gumaiza (*r*_*s*_ = - 0.585), and Hilat Khateir (*r*_*s*_ = - 0.496). Maximum temperature showed positive correlation with black fly counts only in Galabat focus. With the exception of one collection site in Nady village, Abu-Hamed, there was no significance between precipitation and black fly counts in both foci (Table 3).

**Table 3:** Correlation between climatic variables and the adult black flies *Simulium damnosum s*.*l*. counts from Abu-Hamed and Galabat foci during the study periods 2007–2008 and 2009–2010.

Variable	Abu-Hamed		Galabat	
	Nady	Kalasecal	Gumaiza	Hilat Khateir
**Precipitation**	0.489	0.338	0.240	0.096
	(0.015)*	(0.106)	(0.259)	(0.656)
**Maximum Temperature**	-0.357	-0.256	-0.616	-0.690
	(0.087)	(0.228)	(0.001) *	(0.00) *
**Minimum Temperature**	0.799	-0.676	-0.585	-0.496
	(0.000)*	(0.000) *	(0.003) *	(0.014)*

The values in the table represent the correlation coefficient *r*_*s*_ and the *P*-value between brackets

(*) indicate significance

**Fig 6:**
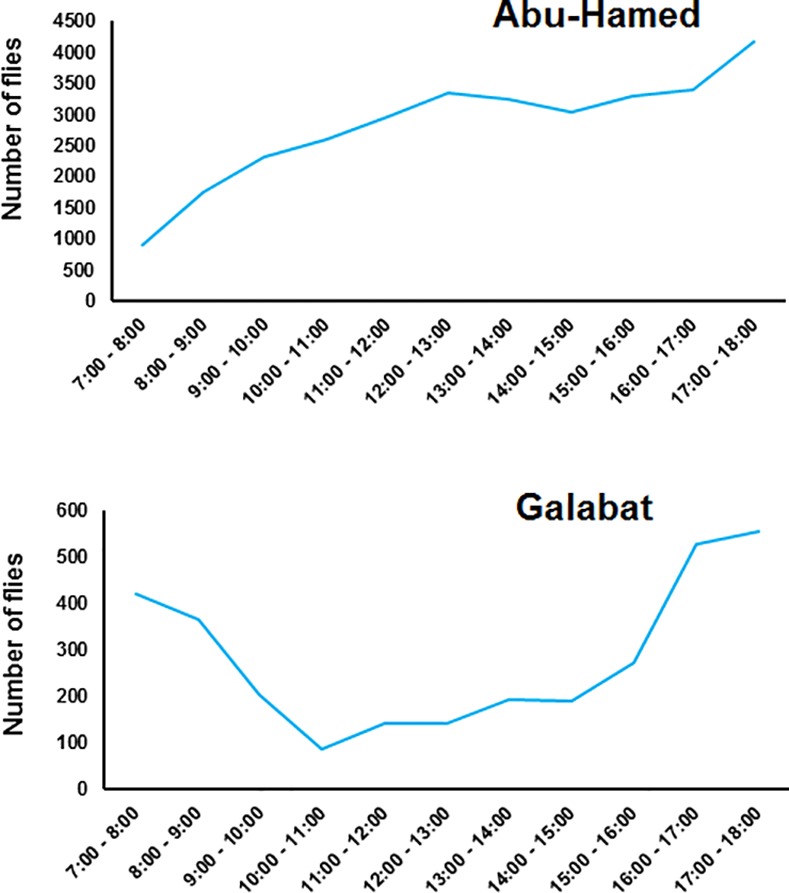
Hourly-based distribution of host-seeking females sampled from human-baits collection in Abu-Hamed and Galabat, Sudan.

The hourly-based distribution of black flies showed a unimodal activity pattern of *S*. *damnosum s*.*l*. in Abu-Hamed focus, where the flies were increased in numbers from the early hours in the morning and reached their peak activity in the later hours of the day (10:00 to 18:00) (Fig 6). Black fly daily activity exhibited a bimodal pattern with an early morning peak (06:00 to 10:00) and a more pronounced late afternoon peak (14:00 to 18:00) in the Galabat focus (Fig 6).

## Discussion

This study examined the seasonal variations of *S*. *damnosum s*.*l*. flies by comparing relative abundance, monthly biting-rate and hourly-based distribution during wet and dry seasons of 2007–2008 and 2009–2010 in four endemic villages in Abu-Hamed and Galabat foci in Sudan. Information about the dynamics of black fly larval populations is considered essential in planning meaningful surveys, in selecting appropriate time schedules for sampling, in studying species distribution and to better understand the local epidemiology of onchocerciasis transmission [31]. Local conditions including rain and high air moisture play an important role in the seasonal changes of black flies [32].

The breeding sites of immature stages of *S*. *damnosum s*.*l*. were found most prevalent in Abu-Hamed as compared to Galabat; this difference in the distribution of the immature stages may result from the water seasonality of Atbara River and less-dense vegetation of plants associated with black fly breeding [23]. Comparison between the two breeding seasons in the two foci showed marked differences in seasonality; the aquatic stages were more abundant in dry season (November-May) in the clean water of River Nile at Abu-Hamed focus, while the aquatic stages were more abundant in the wet season (July-November) in the turbid and clean waters of Atbara River in Galabat focus. The high turbidity of water in the flooding season at Abu-Hamed (August-October) may be a limiting factor in the breeding of *S*. *damnosum s*.*l*. but this might not be the same case in Galabat focus.

The aquatic and adult stages disappeared from July to October in Abu-Hamed and from February to June in Galabat. The high flooding waters of River Nile during these months completely cover black fly breeding sites and may have attributed to the disappearance of the black flies. The Atbara River is reduced to almost a trickle during the dry season and, as such, could only support breeding of black flies in large numbers during the wet season when it starts flowing after the first heavy rains in the Ethiopian highland, and considerable numbers of adult flies were caught even before any larvae or pupae were detected in the river.

The flooding and high rainfall greatly influenced the number and prevalence of the breeding sites and hence the density of black flies in Abu-Hamed and Galabat, respectively. The rainfall causes increase in the speed and nutritive status of the Atbara River, leading to an expansion of larval breeding sites with consequent increases in larval numbers and hence adult fly populations. This increase may be attributed to the stimulus of increased oxygen content of water during the rainy months, which enhances immature black fly stages development and results in an increase in the adult populations [1, 33]. These observations were not the same during Abu-Hamed flooding season. The stormy weather of the Nubian Desert and flooding of River Nile, on the contrary, might be a potential factor for washing away most of the breeding sites, thus resulting in a smaller, almost negligible, fly population.

The monthly biting activity of black flies observed in this study confirmed previous observations on black fly biting behavior in the Abu-Hamed and Galabat [18, 19]. In Abu-Hamed, the maximum biting activity was found during dry season which is characterized by moderate temperatures to the wet flooded season [19]. These observations corresponded to the preference of the black fly to the higher temperatures (Fig 2). The response of the black fly to temperatures was similar to the other haematophagus insects which have shown higher temperatures as the most important stimulus for probing and feeding activity [13].

The highest average black fly biting activity was previously recorded [33] during the dry season with harmattan wind where the biting activities of the flies concentrated in the evenings in Enugu State, Nigeria. We described similar monthly and daily black fly activity pattern in the Galabat focus, which lies in the same savannah zone [18]. In a study in north Cameroon [34], the seasonal variations of the fly populations in the Sudan-savanna area were linked to the water-discharge of the breeding rivers, and the diurnal variations in the biting activity have been related to variations in the temperature, humidity and intensity of light. Relative humidity data could not be obtained for this study.

The current study showed a clear difference in hourly-based biting distribution between the two Sudanese onchocerciasis foci, especially in the wet season. The difference could be attributed to the ecology and microclimate of the two foci [35, 36]. The unimodal peak at Abu-Hamed may be synchronized with farming activity period in the focus, while, the bimodal daily biting pattern hampers human activities in the Galabat area, with morning and late afternoon biting peaks that generally coincide with the active farming periods, washing of clothes, grazing and drinking of livestock, and collection of drinking water in the area. In Nigeria, Opara and his co-authors [1] observed that hourly-biting activity of *S*. *damnosum* is affected by illumination and temperature in Akwa Ibom State. Similarly, the unimodal and bimodal peaks of black fly activity in Abu-Hamed and Galabat foci seem to be synchronized with variations of daily temperature and illumination characterizing climatic conditions in each region. In addition, differences in black fly species composition in Abu-Hamed and Galabat may play a role in the variation seen in black fly daily and seasonal activities. Cytogenetic and molecular studies of black flies in Abu-Hamed showed a newly described from that of savanna type [5] while the cytospeceis of black flies in Galabat is not known yet.

Our study showed remarkable difference between the Abu-Hamed and Galabat foci of Sudan in seasonality, abundance and climatic variables affecting black fly populations. Similar differences were reported in the parasite populations of the two foci, and genetic differences in the human host populations in the two foci have also being suggested [37]. While the Galabat focus is similar in many ways to other savanna foci in west Africa [18], Abu-Hamed focus is unique, being the most northern focus in the world, isolated in the Nubian desert of North Sudan [15, 18, 19].

## Conclusions

The information provided here on human exposure in relation to vector seasonality, distribution and biting activity represents base-line data on local ecological variations of onchocerciasis transmission that could influence the ultimate success of onchocerciasis control/elimination programs and has obvious implications for planning operations in variable ecological situations, especially when vector control is considered in addition to mass treatment activities. Furthermore, this study provides information for the rational development of entomological surveillance systems, which are required to monitor the success of onchocerciasis elimination and guidance to reduce the nuisance of black flies in endemic areas after interruption of transmission of the parasite.

## Supporting Information

S1 Fig Sampling localities and habitat structure in Sudan. (A) Collection of aquatic stages of *S*. *damnosum* from breeding plants in Sudan. (B) Typical breeding site of onchocerciasis vectors. (C) Vegetation and rocks found in fast flowing water in Atbara River, Sudan. (D) Searching for breeding places of onchocerciasis vectors in Galabat focus, Sudan.
(TIF)
