# Supplementary figures and images for: Seasonal Variation in Biting Rates of Simulium damnosum sensu lato, Vector of Onchocerca volvulus, in Two Sudanese Foci
Source: PLoS One. 2016 Mar 4;11(3):e0150309. doi: 10.1371/journal.pone.0150309 (PMC4778939; doi:10.1371/journal.pone.0150309)

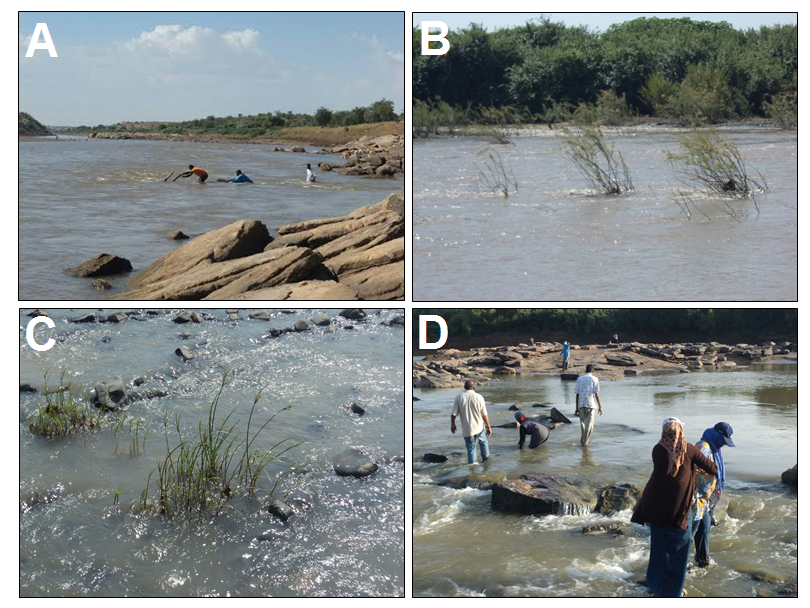

Supplement: S1 Fig — (A) Collection of aquatic stages of S. damnosum from breeding plants in Sudan. (B) Typical breeding site of onchocerciasis vectors. (C) Vegetation and rocks found in fast flowing water in Atbara River, Sudan. (D) Searching for breeding places of onchocerciasis vectors in Galabat focus, Sudan. (TIF) [file pone.0150309.s001.tif]
